# A mechanistic multicentre, parallel group, randomised placebo-controlled trial of mesalazine for the treatment of IBS with diarrhoea (IBS-D)

**DOI:** 10.1136/gutjnl-2015-309122

**Published:** 2015-03-12

**Authors:** Ching Lam, Wei Tan, Matthew Leighton, Margaret Hastings, Melanie Lingaya, Yirga Falcone, Xiaoying Zhou, Luting Xu, Peter Whorwell, Andrew F Walls, Abed Zaitoun, Alan Montgomery, Robin Spiller

**Affiliations:** 1 NIHR Nottingham Digestive Diseases Biomedical Research Unit, University of Nottingham, Nottingham, UK; 2 Nottingham Clinical Trials Unit, University of Nottingham, Nottingham, UK; 3 Neurogastroenterology Unit, Wythenshawe Hospital, Manchester, UK; 4 Immunopharmacology Group, University of Southampton, Southampton General Hospital, Southampton, UK; 5 FRAME laboratory, University of Nottingham, Nottingham, UK; 6 Immunopharmacology Group, University of Southampton, Southampton General Hospital, Southampton, UK; 7 Department of Histopathology, Nottingham University Hospital Trusts, Nottingham, UK

**Keywords:** DIARRHOEA, IRRITABLE BOWEL SYNDROME, 5-AMINOSALICYLIC ACID (5-ASA)

## Abstract

**Introduction:**

Immune activation has been reported in the mucosa of IBS patients with diarrhoea (IBS-D), and some small studies have suggested that mesalazine may reduce symptoms. We performed a double-blind, randomised placebo-controlled trial of 2 g mesalazine twice daily versus placebo for 3 months in patients with Rome III criteria IBS-D. Primary outcome was daily average stool frequency during weeks 11–12; secondary outcomes were abdominal pain, stool consistency, urgency and satisfactory relief of IBS symptoms.

**Methods:**

Participants were randomised after a 2-week baseline stool diary. All participants completed a 12-week stool diary and at the end of each week recorded the presence of ‘satisfactory relief of IBS symptoms’.

**Results:**

136 patients with IBS-D (82 women, 54 men) were randomised, 10 patients withdrew from each group. Analysis by intention to treat showed the daily average stool frequency during weeks 11 and 12 were mean (SD), 2.8 (1.2) in mesalazine and 2.7 (1.9) in the placebo group with no significant group difference, (95% CI) 0.1 (−0.33 to 0.53), p=0.66. Mesalazine did not improve abdominal pain, stool consistency nor percentage with satisfactory relief compared with placebo during the last two-weeks follow-up.

**Conclusions:**

This study does not support any clinically meaningful benefit or harm of mesalazine compared with placebo in unselected patients with IBS-D. More precise subtyping based on underlying disease mechanisms is needed to allow more effective targeting of treatment in IBS.

**Trial registration number:**

NCT01316718.

**Significance of this study**

**What is already known on this subject?**

- Treatment for IBS with diarrhoea (IBS-D) is limited and normally based on symptom control.
- Ongoing ‘immune activation’ in the gut mucosa of patients with IBS-D.
- Mesalazine may be beneficial for the treatment of IBS-D symptoms.

**What are the new findings?**

- Mesalazine 4 g/day was no better than placebo in relieving symptoms of abdominal discomfort or disturbed bowel habit.
- Mesalazine did not reduce mast cell percentage area stained.
- Raised stool calprotectin level was associated with less psychological distress implying a more gut-centred abnormality.

**How might it impact on clinical practice in the foreseeable future?**

- A subgroup of patients with postinfectious IBS may benefit from mesalazine.

## Introduction, background and objectives

IBS is a heterogeneous condition seen commonly in both primary and secondary care in the UK, where it accounts for 3%^1^ and 40% of all consultations respectively,^2^ a process that consumes considerable medical resources. This chronic condition also impacts on patients’ quality of life and their performance at work and home.^3^ ^4^ The diarrhoea subtype particularly impairs quality of life by limiting patients’ diet and the ability to travel or eat out.^5^ Around two-thirds of patients with IBS show anxiety or depression and multiple somatic symptoms,^6^ but how much is cause and how much is effect of the distressing symptoms remains unclear and likely varies from case to case. IBS with diarrhoea (IBS-D) may develop after inflammation due to bacterial gastroenteritis (postinfectious IBS (PI-IBS))^7^ in whom the immune response can be prolonged.^8^ Recent studies have also shown ‘immune activation’ in the mucosa of patients with IBS-D without an infectious origin. Increased numbers of mast cells and release of mast cell mediators such as mast cell tryptase, serotonin and histamine have been reported in some^9–11^ but not all series.^12^ Other immune cells such as T lymphocytes and serotonin (5-hydroxytryptamine (5-HT)) containing enterochromaffin cells have been reported as increased in PI-IBS.^13^ Numerous recent studies have suggested impaired mucosal barrier in IBS,^14^ which by allowing access of luminal bacterial products to the mucosal immunocytes might cause immune activation.^15^ These studies suggested that an anti-inflammatory treatment might be beneficial, an idea that has been supported by some small pilot studies that appeared to show improvement in abdominal pain, stool frequency and consistency, especially in patients with PI-IBS.^16^ ^17^ One study showed a reduction of mast cells following treatment of mesalazine in 10 unselected patients with IBS,^18^ but like the other studies, it was too small to be sure of its significance. Our aim was therefore to assess the effect of mesalazine as a treatment for IBS-D and to also assess its impact on mast cell numbers and mediator release in an attempt to predict treatment response.

## Methods

### Trial design

**Figure 1:**
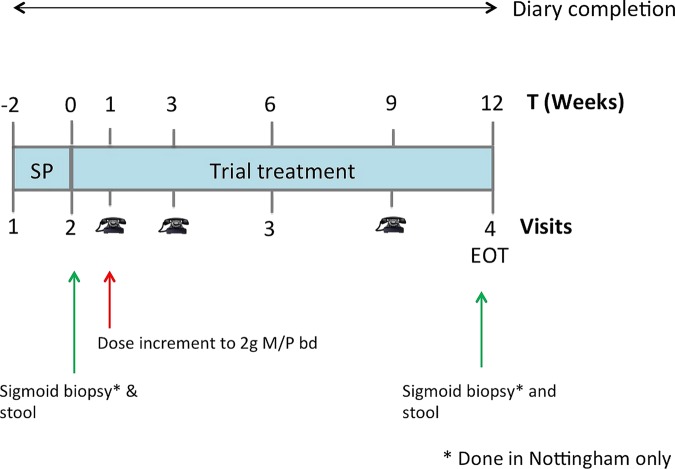
Study design. This shows the timeline for the study and the 12-week treatment period during which participants were randomised to receive either mesalazine or placebo. bd, twice daily; EOT, end of trial; 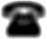, telephone visits.

This was a multicentre, two-arm, parallel group, double-blind, randomised placebo-controlled trial comparing mesalazine with placebo in patients with IBS-D. Eligible participants were randomised to receive either mesalazine or placebo 2 g once a day for a week, and if they tolerated the dose, it was increased to 2 g twice a day for 11 weeks. There were four visits altogether (weeks −2, 0, 6 and 12) and telephone visits in between (weeks 1, 3 and 9) to ensure tolerance and compliance with medication (figure 1). Participants were required to complete weekly stool diaries for 12 weeks. We collected stool and sigmoid biopsy samples before and at the end of trial (EOT) to look for biomarkers, but for practical reasons this was only performed on those patients recruited in Nottingham. The trial was registered on clinicaltrials.gov (identifier NCT01316718) and European Union clinical trials register with EudraCT number 2010-018340-14. Initial recruitment into this trial was slow, and it was felt that the eligibility criteria for IBS-D were too demanding. We therefore modified the eligibility criteria for IBS-D (see section ‘Participants’) following registration with the clinicaltrials.gov to reflect the fact that, as others have found, the bowel habit of patients with IBS-D is less abnormal than patients' recall suggests.^19^

### Randomisation and blinding

This was a double-blind parallel group study. The participant, supervising doctor and/or study nurse were unaware of the treatment allocation. The randomisation was based on a computer-generated pseudo-random code using random permuted blocks of randomly varying size, created by the Nottingham Clinical Trials Unit (NCTU) in accordance with their standard operating procedure and held on a secure server. Allocation was stratified by the recruiting centre. The supervising doctor or study nurse obtained a randomisation reference number for each participant by means of a remote, internet-based randomisation system developed and maintained by NCTU. The sequence and decode of treatment allocations were concealed until all interventions were assigned and recruitment, data collection and all other trial-related assessments were completed and study files locked. The NCTU Data Manager and the Nottingham University Hospitals Trust Trials pharmacy had access to the treatment allocation. All data collected were entered into a secured online database set up by NCTU.

A trial management group was set up to oversee the operational aspects of the trial. The group met regularly to review progress of the trial and address any urgent issues. The Data Monitoring and Ethics committee undertook the safety monitoring functions of the trial and provided recommendations to the trial steering committee over the course of the trial. A trial steering committee was set up to ensure the study was well executed.

### Sample size calculation

Our previous study on patients with IBS-D gave a mean stool frequency of 3.1 (SD 2.0). Tuteja and colleagues reported mesalazine decreasing stool frequency by 1.4 bowel movements per day.^20^ Our study had 80% power to detect such an effect at the 1% two-sided α level.

Much smaller numbers were needed to assess the effect of mesalazine on mast cell numbers and tryptase release. Corinaldesi *et al*^18^ reported a 36% decrease in mast cell numbers from mean 9.2 (SD 2.5), which requires just 16 patients in each group to show such a decrease with a power of 90% at the 5% α level. We therefore decided to assess the biopsies on patients recruited in Nottingham only in order to ensure uniform processing.

We aimed to randomise at least 125 patients to allow for 20% dropout rate, but owing to recruitment ongoing at multiple sites and patient requests, we actually recruited 136.

### Participants

Patients with IBS-D were recruited from gastroenterology clinics at the Nottingham University Hospitals, seven other secondary care hospitals in the UK and via the Trent Primary Care Research Network from 1 April 2011 to 31 May 2013. The patients were required to meet the modified Rome III criteria for IBS-D,^21^ defined as a stool frequency of ≥3/day for >2 days/ week and ≥25% of stools to be of type 5–7 and ≤25% type 1–2 according to the Bristol Stool Form Scale (BSFS),^22^ as assessed from their screening stool diaries. The unmodified Rome III criteria require ≥25% of stools to be of type 6–7, but we found this excluded around one-third of otherwise typical patients who met Rome III criteria based on recall, hence the modification. To exclude other causes of diarrhoea, we required normal colonoscopy and colonic biopsies, normal full blood count, serum calcium and albumin, C-reactive protein and negative serological test for coeliac disease. Lactose intolerance was tested by asking patients to consume 568 mL (1 pint) of milk after an overnight fast and performing a lactose breath hydrogen test if they developed pain or diarrhoeal symptoms within 3 h. If the stools were watery and frequent, patients underwent a 7-day retention of selenium^75^-labelled homocholic acid taurine test or a trial of cholestyramine to exclude bile acid malabsorption. If any of these tests were positive, patients were excluded from the study. Most patients have continued to be followed up, and no new diagnoses have emerged. Other inclusion criterion was age 18–75 years. Exclusion criteria were prior history of major abdominal surgery, liver or kidney impairment or chronic ingestion of any anti-inflammatory drugs or medications that could affect the gut motility. All childbearing female patients tested negative on the pregnancy test during the randomisation day and had to agree to adequate contraception during the trial. Patients who were on long-term selective serotonin reuptake inhibitors or tricyclic antidepressants were included if they were on a stable dose for 3 months and willing to keep the dose unaltered throughout the trial. During the screening period of 2 weeks, patients were only allowed a maximum of two doses of 4 mg loperamide per week. Once randomised, patients were allowed to take loperamide to control their symptoms as we hypothesised that mesalazine would take at least six weeks to exert its effect on the gut, assuming that it acts by altering the immunocytes within the mucosa. However, during the last two weeks of the trial when the bowel movement endpoints were assessed, the patients were not allowed any loperamide or antibiotics.

### Investigational medical product

The mesalazine used for this study was a licensed slow release granule formulation of 2 g (PENTASA, Ferring Pharmaceuticals) and a matching placebo granule formulation (QPharma AS, Sweden). In order to maintain blinding, active and placebo granules were packed in matching, unidentified, trial-specific foil sachets (Ferring Pharmaceuticals). Newcastle Specials at Newcastle-upon-Tyne Hospital National Health Service Foundation Trust carried out final labelling and release of blinded trial treatment packs. All sites involved had appropriate licences in place.

### Data collection

Baseline demographics were collected at visit 1 (screening visit) including age, gender and ethnicity. Data for Hospital and Depression Scale^23^ (HADS) and score from the Patient Healthy Questionnaire 15^24^ (PHQ15) were collected at visit 2, after randomisation and at final visit 4 (EOT). We used a 7-day weekly stool diary throughout the trial to provide information on the stool form based on BSFS, abdominal pain severity, urgency of defecation and abdominal bloating. The latter three symptoms were scored between 0 (no symptom) and 10 (extremely severe).

### Compliance

Compliance was defined a priori as taking ≥75% of the medication throughout the 12 weeks. Each patient was given two boxes of medication during the 12-week study, each box containing 100 sachets. The amount of medication taken was calculated from the number of medication sachets returned at EOT. Compliance with medication and baseline characteristics of compliers (defined as taking >75% of the medication throughout the 12 weeks) and non-compliers were similar in both groups (see online supplementary table S5).

### Outcomes

#### Clinical outcomes

The primary clinical outcome was stool frequency in the last two weeks of trial follow-up (weeks 11 and 12). Secondary outcomes were abdominal pain, urgency and stool consistency. These were averaged over the last two weeks of the trial follow-up. At the end of each week, patients were asked, “Have you had satisfactory relief of your IBS symptoms this week?”. Satisfactory relief of IBS symptoms at EOT was defined as answers to ‘yes’ on both weeks 11 and 12 of stool diary. Information provided for <10 of 14 days of stool diary was recorded as missing. All completed an assessment of anxiety and depression (Hospital Anxiety and Depression Scale), and multiple somatic symptoms were recorded using the Patient Health Questionnaire-12 Somatic Symptom Scale (PHQ12-SS).^6^

#### Mechanistic outcomes

The primary mechanistic endpoint was to assess the mast cell numbers from the percentage area stained at week 12. The secondary endpoint was to assess mast cell activation in biopsy supernatants and to relate these to symptoms. Mast cell activation was defined as elevation of any mediator component including mast cell tryptase, chymase, carboxypeptidase A3 and/or histamine.

#### Stool samples and sigmoid colon biopsies

These were collected at week 0 and EOT from patients recruited in Nottingham. Stools collected were analysed for calprotectin. A commercially available calprotectin ELISA kit (Buhlmann, Schönenbuch, Switzerland) was used for extraction and quantification of stool calprotectin. Normal range for stool calprotection is defined as <50 μg/g.

Sigmoid biopsies were collected for immunohistochemistry for mast cell tryptase, CD3, CD68 and 5-HT. Tissues were processed and stained in the histopathology laboratory in Nottingham University Hospitals Trust, UK. While other cells were individually counted per mm^2^ mast cell, numbers were assessed from the percentage area stained in the area of interest as this was felt to more accurately reflect mast cell activity by including mast cells, which were degranulating and hence indistinct. A further set of biopsies was maintained in culture, and supernatants collected were assayed for mast cell tryptase, chymase, carboxypeptidase A3 and histamine. The biopsy tissues were incubated immediately in Hanks’ medium at 37°C, 5% CO_2_ for 30 min before storing at −80°C until assays for mast cell mediators were performed by the Immunopharmacology Group at the University of Southampton.

See supplementary file for details of methods for immunohistochemical staining for mast cell tryptase, CD3, CD68 and 5-HT staining and measurement of mast cell mediator release.

### Statistical methods

Analysis and presentation of data was in accordance with Consolidated Standards of Reporting Trials guidance, using Stata V.13. Balance between the trial arms at baseline was examined using appropriate descriptive statistics.

The general approach for between-group comparisons was to analyse participants according to allocation without imputation of missing data. The primary data set comprised participants with completed stool diary for at least 10 days out of 14. We used a generalised linear mixed model to compare mesalazine group and placebo group for the primary outcome, with adjustment for the baseline value of the outcome, and study centre as a random effect. Additionally, we adjusted for any variables showing imbalance at baseline in secondary models. We compared the characteristics of participants who did and did not adhere to the study medication before estimating the treatment effect if the medication was actually taken using complier average causal effect (CACE) analysis. We investigated the effect of missing primary outcome data using multiple imputation. The secondary outcomes were assessed using similar models as for primary outcome, or logistic or Poisson regression as appropriate dependent on outcome type.

We conducted a number of prespecified subgroup analyses for each of the following three outcomes: (1) stool frequency during weeks 11–12, (2) number of days with any stool consistency scoring 6 or 7 during weeks 11–12 and (3) mean score of worst pain for each day averaged over weeks 11 and 12. We investigated whether there were any differences in between-group effects according to the following baseline variables: (1) anxiety, (2) stool frequency, (3) abdominal pain and (4) mast cell activation. These subgroup analyses were conducted by including appropriate interaction terms in the regression models, and as the study was not powered on the basis of detecting any such subgroup effects, these are considered exploratory and would require confirmation in future research.

The primary mechanistic hypothesis to be investigated was that treatment with mesalazine reduces inflammation, which in turn reduces clinical symptoms. The aim of this type of analysis is to estimate how much of any observed treatment effect can be attributed to a variable that is thought to be an intermediate on the causal pathway, or mediator. After summarising inflammatory markers at baseline and 11–12 weeks’ follow-up by trial arm using appropriate descriptive statistics, we examined change in these markers (stool calprotectin, mast cell tryptase, mast cell percentage area stained) and change in stool frequency using scatterplots.

## Results

### Patient flow

**Figure 2:**
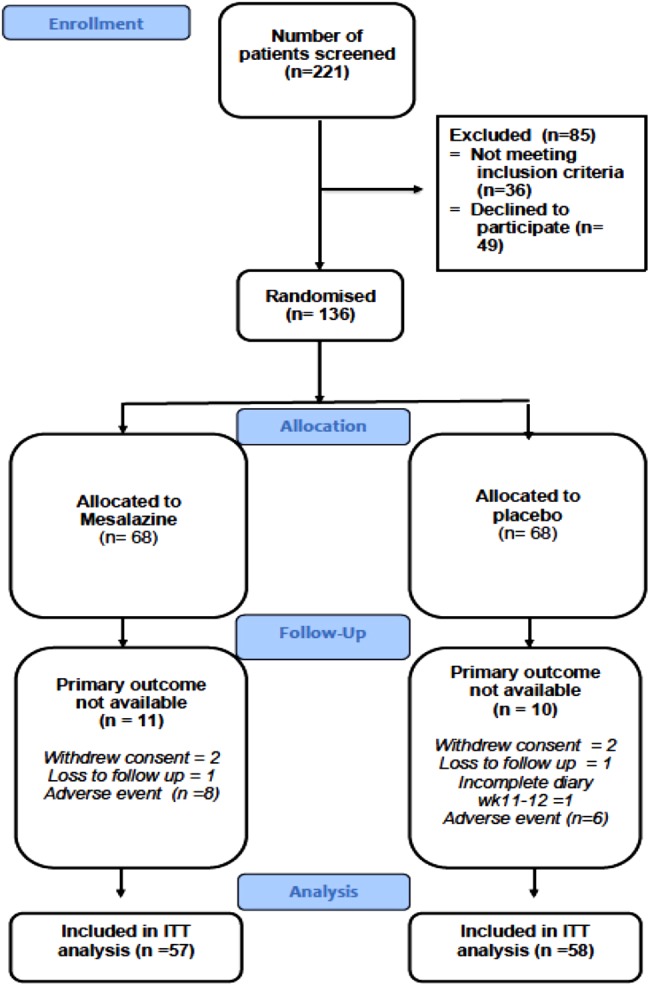
Participant flow in the study. ITT, intention to treat.

A total of 221 patients were screened for this trial, of whom 85 were excluded from the study and 136 were randomised 1:1 to receive either mesalazine or placebo (figure 2). The commonest reasons for exclusion were (a) failure to meet the required severity of diarrhoea on the 2-week diary during the initial 2-week screening period and (b) patients not able to commit their time to the study due to the multiple visits to hospital. In total, 116 patients completed the study, of whom 115 (57 treatment and 58 control) had primary outcome data available. One patient from the placebo group did not complete the stool diary at 11–12 weeks and was therefore excluded. The number of days with stool diary entered at baseline and at EOT was similar (see online supplementary table S7). There were no differences in baseline characteristics between patients who dropped out (non-completer) and those who completed the study (completer) (see online supplementary table S2).

Baseline characteristics of randomised patients in both treatment groups were similar at baseline (table 1). All except four participants were of Caucasian ethnicity.

**Table 1:** Baseline characteristics of randomised patients

Characteristic	Mesalazine (N=68)		Placebo (N=68)	
Age at enrolment: mean (SD)	42.6 (15.2)		47.1 (13.5)	
Gender N (%)	Male 26 (38.2%)		Male 28 (41.2%)	
	Female 42 (61.8%)		Female 40 (58.8%)	
Daily average stool frequency Mean (SD)	3.6 (1.6)		3.6 (1.8)	
Daily mean abdominal pain score Mean (SD)	4.1 (2.2)		3.6 (2.0)	
Number of days with urgency Median (IQR)	13 (10,14)		12 (9,14)	
Stool consistency Mean (SD)	5.4 (0.7)		5.6 (1.0)	
Hospital anxiety and depression score				
Anxiety score Mean (SD)	9.1 (4.5)		8.6 (4.3)	
Depression score Mean (SD)	5.6 (4.2)		5.0 (3.3)	
PHQ12-SS score Mean (SD)	7.8 (4.5)		8.2 (5.2)	

PHQ12-SS, Patient Health Questionnaire-12 Somatic Symptom Scale.

### Clinical outcomes

#### Primary outcome

There was no evidence of any difference between Mesalazine and placebo treatment in reduction of daily average stool frequency. The daily average stool frequency at EOT (weeks 11 and 12) was similar between the two groups, with mean (SD) of 2.8 (1.2) in Mesalazine and 2.7 (1.9) in placebo groups, and adjusted between-group difference (95% CI) of 0.1 (−0.33 to 0.53), p=0.66. Additional adjustments for variables (age, abdominal pain score, number of days with urgency and PHQ15 score) displaying imbalance at baseline did not materially change the results. Sensitive analysis using multiple imputation of missing data did not show any difference in daily average stool frequency between Mesalazine and placebo with adjusted difference in mean frequency (95% CI) of 0.06 (−0.18 to 0.99), p=0.17.

### Compliance

Analysis of the primary outcome using CACE approach showed no difference between the two treatment arms with mean difference (95% CI) of 0.2 (−0.6 to 0.9).

Preplanned subgroup analysis of the primary outcome by baseline daily average stool frequency suggested that mesalazine may be more effective among patients with greater baseline stool frequency (table 2). The adjusted interaction coefficient was −0.26, 95% CI −0.51 to −0.01, p=0.04. There was no evidence of any subgroup effects according to baseline abdominal pain (p=0.36) or baseline HADS (p=0.79).

**Table 2:** Subgroup analysis based on baseline daily average stool frequency

	Mesalazine (N=57)	Placebo (N=58)
Daily mean stool frequency at 11–12 weeks by baseline frequency, mean (SD)		
Baseline frequency ≤2.4	1.7 (0.4)	1.6 (0.5)
Baseline frequency >2.4 and ≤3.4	2.2 (0.5)	2.2 (1.1)
Baseline frequency >3.4 ≤4.6	3.1 (1.3)	2.7 (0.9)
Baseline frequency >4.6	4.1 (1.1)	4.7 (2.9)

#### Secondary endpoints

There was no evidence of any differences between the groups in clinical symptoms such as abdominal pain severity, average stool consistency and number of days with stool consistency type 6–7 (table 3). There was strong evidence that mesalazine treatment increased the number of days with urgency by about 20%. There was no effect on the HADS score and somatic symptom score PHQ12-SS following treatment of mesalazine compared with placebo (table 3).

**Table 3:** Clinical secondary endpoint results

	EOT (11–12 weeks)	Between group comparison at 11–12 weeks (95% CI)	p Value
Average abdominal pain score, mean (SD)			
Placebo	2.2 (2.1)	–	–
Mesalazine	2.8 (2.1)	–	–
Mesalazine vs placebo	–	0.07 (−0.54 to 0.68)	0.83
Number of days with urgency, median (IQR)			
Placebo	8 (1–13)	–	–
Mesalazine	11 (5–14)	–	–
Mesalazine vs placebo	–	1.22 (1.07 to 1.39)*	0.003
Average stool consistency, mean (SD)			
Placebo	4.7 (1.1)	–	–
Mesalazine	4.7 (1.0)	–	–
Mesalazine vs placebo	–	0.13 (−0.21 to 0.48)	0.45
Number of days with consistency score 6 or 7, median (IQR)			
Placebo	6 (2–9)	–	–
Mesalazine	7 (2–11)	–	–
Mesalazine vs placebo	–	1.09 (0.95 to 1.27)	0.21
Number of people with satisfactory relief of IBS symptoms, n (%)			
Placebo	24 (40.7%)	–	–
Mesalazine	25 (43.9%)	–	–
Mesalazine vs placebo	–	1.13 (0.51 to 2.47)†	0.76
Mean HADS anxiety score			
Placebo	6.9 (3.6)	–	–
Mesalazine	7.5 (5.0)	–	–
Mesalazine vs placebo	–	0.67 (−0.38 to 1.72)	0.21
Mean HADS depression score			
Placebo	3.7 (3.2)	–	–
Mesalazine	4.7 (5.1)	–	–
Mesalazine vs placebo	–	0.49 (−0.41 to 1.39)	0.29
Mean PHQ12-SS score (mean(SD))			
Placebo	5.7 (3.9)	–	–
Mesalazine	6.2 (4.4)	–	–
Mesalazine vs placebo	–	0.49 (−0.76 to 1.74)	0.45

*Incident rate ratio. †OR.

EOT, end of trial; HADS, Hospital and Depression Scale; PHQ12-SS, Patient Health Questionnaire-12 Somatic Symptom Scale.

### Mechanistic outcomes

#### Primary endpoint

**Figure 3:**
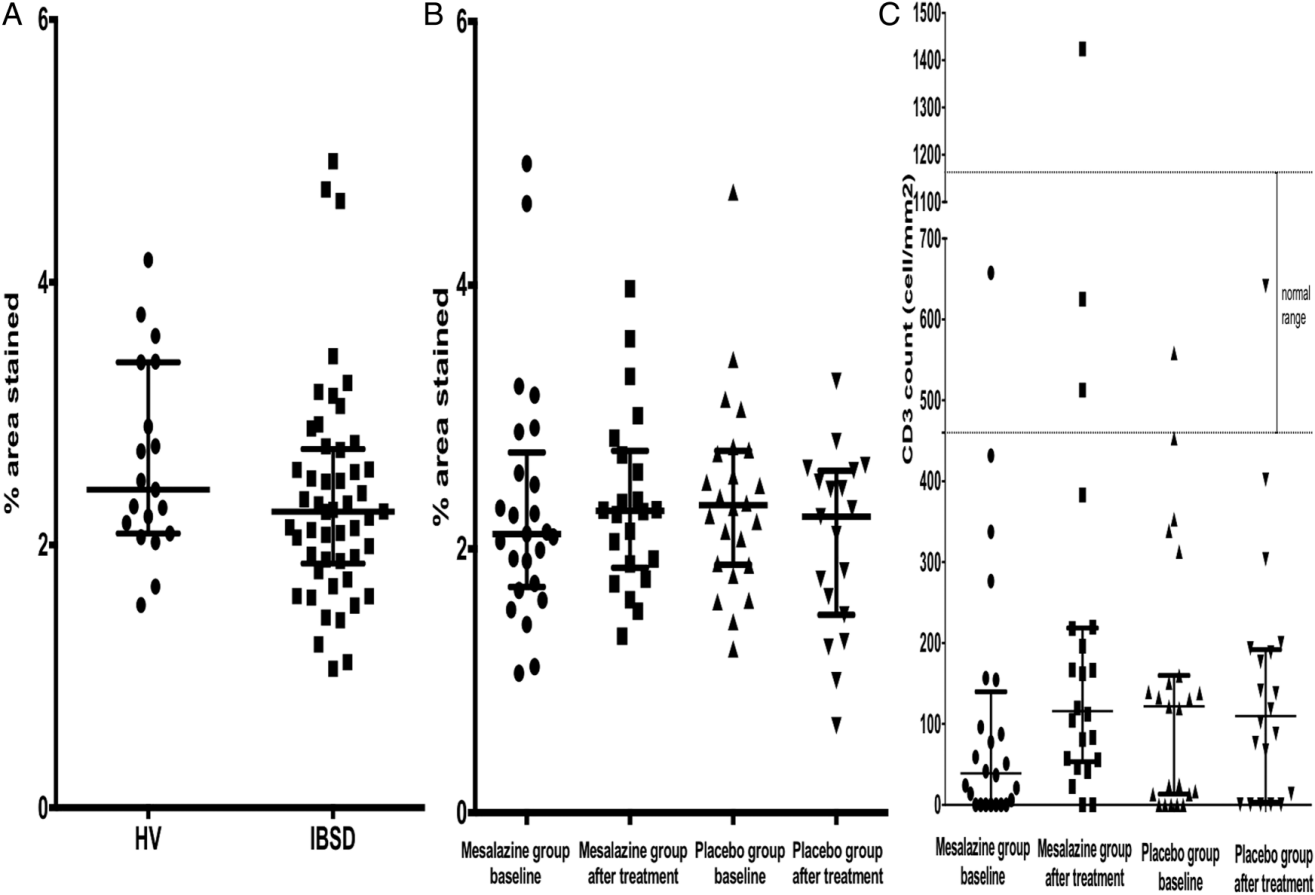
(A) Baseline mast cell percentage area stained in patients with IBS with diarrhoea (IBS-D) and healthy controls (HV); (B) mast cell percentage area stained before and after mesalazine or placebo groups; (C) CD3-positive cells before and after treatment with mesalazine or placebo.

Initial baseline mast cell assessments were compared with 21 healthy controls in a study performed previously using an identical protocol^25^ and 49 patients with IBS-D in this study. Due to patient withdrawals, there were 41 biopsy samples, before and after treatment, available for analysis (22 mesalazine, 19 placebo). The baseline mast cell percentage area stained were similar between patients with IBS-D and the healthy controls giving a median (IQR) of 2.25 (1.86–2.73) vs 2.42 (2.09–3.39) % (figure 3A). Following treatment with mesalazine, there was no significant change in mast cell percentage area stained compared with placebo. The mean difference (SD) in mast cell percentage area stained following treatment with mesalazine was 0.09 (0.55) and with placebo was −0.19 (0.76) % (figure 3B).

#### Secondary endpoints

There was no correlation between mast cell percentage area stained and clinical symptoms of average abdominal pain severity, bloating, urgency, average stool frequency and average stool consistency. There was no significant association between mast cell percentage area stained in biopsy tissue and supernatant levels of tryptase, chymase, carboxypeptidase A3 or histamine (see online supplementary table S3).

#### Immune cells

There was no effect of either treatment on 5-HT containing enterochromaffin and CD68 cell numbers. Surprisingly, there was significant increase in CD3 count in the mesalazine group at EOT (figure 3C) with mean difference (SD) CD3 count of 140 (349) /m^2^ compared with placebo 33.1 (153) /m^2^.

#### Stool calprotectin

A subgroup of patients from the Nottingham site provided stool samples for calprotectin analysis. A total of 55 stool samples were collected at the randomisation visit and 53 samples at the EOT visits. In total, 23 stool samples (before and after treatment) were from the mesalazine group and 30 stool samples (before and after treatment) were from the placebo group. Baseline calprotectin level was 59 (19–113) μg/g. There was no significant change in stool calprotectin levels following treatment with mesalazine. Mean differences (SD) were −12.2 (82.7) for mesalazine and 0.1 (87.1) for placebo, p=0.99. There was a weak inverse correlation between baseline calprotectin level with total hospital and anxiety scores, Spearman r=−0.25, p=0.07. We performed a post hoc explanatory analysis dividing patients according to their baseline calprotectin levels into two groups. Group A (n=14) had calprotectin levels >100 μg/g, and group B (n=41) had calprotectin levels ≤100 μg/g. The total HAD score was significantly lower in group A compared with group B with median (IQR) of 7 (4–14) and 13 (7–18), respectively, p=0.03 (see online supplementary figure S1).

#### Postinfectious IBS

**Figure 4:**
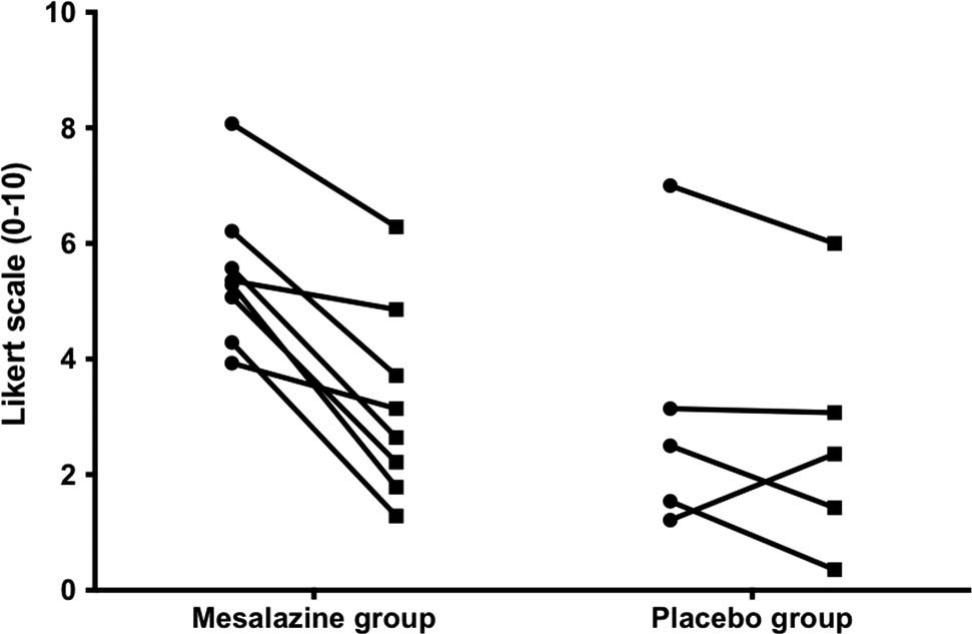
Improvement of abdominal pain severity following treatment of mesalazine in patients with post-infectious IBS.

Thirteen patients with IBS-D from the study fulfilled the criteria for PI-IBS.^13^ The mean stool frequency for PI-IBS and the remaining patients with IBS-D were similar, giving a mean (SD) of 4.25 (2.35) and 3.59 (1.70), respectively, p=0.48. Eight were on mesalazine treatment and five on placebo. Their baseline characteristics were similar (see online supplementary table S4). Following treatment with mesalazine but not placebo, there seemed to be significant improvement in average abdominal pain severity, average urgency score and average daily stool consistency (see figure 4 and online supplementary figure S2).

### Adverse events

The most frequently occurring side effect was exacerbation of IBS symptoms, mostly worsening abdominal pain or diarrhoea. Two (3%) from mesalazine and three (5%) from placebo group complained of this and were withdrawn from the study. Other less frequent side effects are listed in online supplementary table S6. One patient was pregnant in the middle of trial period, although she had a negative pregnancy test at the start of the trial. She was withdrawn from study with no adverse consequence to herself or her newborn.^26^ One patient from the mesalazine group was found to have breast cancer, and she was withdrawn from the study as her IBS symptoms and stool diary would be very difficult to interpret. All participants who developed these adverse events were withdrawn from the study, and their symptoms settled on follow-up.

## Discussion

Our study is one of the largest trials so far looking at the treatment of mesalazine in patients with IBS-D following best practice to ensure that both investigators and patients were blinded to the study and that data analysis was carried out by independent statisticians. We analysed the effect of mesalazine only after 12-weeks treatment as we felt that mesalazine was a disease-modifying treatment rather than symptomatic treatment and early reports suggested benefit was most obvious after 2–3 months.^27^

Our study showed that mesalazine did not improve bowel frequency after 12-weeks treatment compared with placebo in unselected patients with IBS-D. As with other studies in IBS, we found a strong placebo effect on bowel symptoms and also on the total Hospital Anxiety and Depression and Somatic scores (PHQ12-SS), suggesting that patients felt better in general after taking part in the trial. We found no evidence that the improvement in HADS or PHQ12-SS correlated with changes in bowel habit, suggesting these are independent features and not causally related.

Despite lack of benefit in unselected patients, we had a preplanned subgroup analysis of the primary outcome of stool frequency in patients divided according to severity. This suggested that a group of patients who had the greatest bowel frequency did benefit from mesalazine. Our clinical findings seem consistent with another recent report.^28^ There was no significant improvement in other IBS symptoms such as abdominal pain, bloating and stool consistency. There is strong evidence from our study that mesalazine treatment increases the number of days with urgency by about 20%. There have been previous case studies reported of mesalazine worsening diarrhoea in colitis.^29^ ^30^ This may represent an allergic response to the drug as we did find an increase in T lymphocytes.

Raised mast cell numbers in the gut mucosa have been implicated in all subtypes of IBS^31^ but mainly in IBS-D. Mast cells contain many mediators including histamine, serotonin and proteases such as tryptase.^10^ Recently, there has been an interest in tryptase from both mast cells and endogenous pancreatic secretion^32^ as it has been shown to activate proteinase-activated receptor 2, which is found on afferent nerves and can lead to increased sensitivity of bowel distension.^33^ In our study, the mast cell percentage area stained in patients with IBS-D was not elevated compared with those in our previously studied healthy subjects. We were not able to confirm the gender difference in mast cell count of patients with IBS-D previously described by others,^34^ nor did we find any gender effect on other immune cells such as CD3, CD68 and 5-HT containing enterochromaffin cells.

Similarly, the supernatant tryptase levels in patients with IBS-D were not significantly elevated compared with healthy control. Median (IQR) tryptase levels for IBS-D versus healthy control were 4.3 (1.8–8.9) and 6.7 (3.8–11.4) ng/mL, p=0.07. Surprisingly, supernatant histamine levels in our study were lower in patients with IBS-D compared with healthy control, being mean (SD), 0.7 (0.6) and 1.1 (0.8) ng/mL, respectively, p=0.02. There was no correlation between baseline tryptase/histamine/chymase/CPA3 with clinical symptoms. Supernatant levels of tryptase and histamine were not altered following treatment of mesalazine. We found no apparent association between mast cell percentage area stained and supernatant levels of the mast cell mediators examined whether those released by all mast cells (tryptase, histamine) or restricted to a subpopulation (chymase, carboxypeptidase A3). This suggests that the overall degree of mediator release from colonic mast cells is independent of mast cell numbers and factors other than mere numbers determine mediator release. We were not able to confirm either increased mast cell numbers nor increased mast cell tryptase release from biopsies as some^9^ ^10^ but not all^12^ investigators have found. Our study provides no support for the previous suggestion that mesalazine can reduce mast cell numbers.^18^

Stool collected in Nottingham was used to obtain calprotectin level at baseline and EOT. Although the normal calprotectin recommended by the commercial laboratory was <50 μg/g, there is still an uncertainty with patients who have borderline results (50–150 μg/g) as most of them do not have inflammatory bowel disease.^35^ In this study, there were 28 patients with IBS-D who have calprotectin levels >50 μg/g (median (IQR)=105.5 (73.5–173.1)). On repeated testing (placebo group n=16), approximately 44% of the calprotectin levels had normalised after 12 weeks of placebo treatment, a feature others have noted in a series of patients with IBS with intermediate calprotectin levels (50–100 μg/g) subjected to repeated testing.^35^ For all patients who were recruited into the study, we have excluded organic diseases such as inflammatory bowel disease in gastroenterology clinics using standard tests like normal haematology, biochemical and ileocolonoscopy prior to them entering the study. Furthermore, patients have had continued follow-up as outpatients and no new diagnoses have emerged. Others have also reported up to a quarter of patients with IBS have marginally elevated calprotectin though the origin of this is unclear.^36^ ^37^ Interestingly, the subgroup of patients (group A) who had raised calprotectin level (>100 μg/g) have significantly less psychological distress than the group with stool calprotectin level ≤100 μg/g (group B). We speculate that in subgroup A symptoms are secondary to occult local gut inflammation while subgroup B's symptoms are driven primarily by distress, which causes gut symptoms secondarily. Unfortunately, numbers were too small to answer the question of whether subgroup A responded better to mesalazine. Stool calprotectin could therefore be used as a screening tool to allow more detailed studies of the mucosa in IBS-D in the future.

One uncontrolled study has suggested that mesalazine might be effective in treating patients with PI-IBS,^16^ but the only randomised controlled trial of mesalazine in this condition was negative, though possibly underpowered.^17^ In our post hoc analysis, a small subgroup fulfilling criteria for PI-IBS appeared to benefit from mesalazine, but our study was also underpowered. Interestingly, the study of the outbreak of enterohaemorrhagic *Escherichia coli* O104:H4 infection in Germany suggests that mesalazine treatment substantially reduced the incidence of PI-IBS,^38^ which further supports this idea that a larger and more adequately powered study specifically focused on PI-IBS would be worthwhile.

Although mesalazine has been available to use for many decades with good safety profile, our adequately powered study has showed it does not help the majority of patients with IBS-D. The fact that certain subgroups might benefit emphasises that there is still a need for better phenotyping of this heterogeneous group of patients when evaluating new treatments.

### Limitations

Despite strict entry criteria, our population was still heterogeneous. In retrospect, we would have been better if we had stratified by postinfectious onset. We did consider this but felt that this would make the trial very difficult to recruit to. We could overcome this in future studies by having a great many more recruitment sites and screening around five times as many participants, given that PI-IBS accounts for only around 20% of all cases of IBS-D, but this would require more resources than we had available to us. It is worth noting that there is an appreciable loss to follow-up (15.5%) but not out of line with other similar IBS studies. Dropouts are mostly likely due to failure of treatment and so unlikely to account for our negative result.

### Research recommendations

1. Our data suggest that it is unlikely that future trials of mesalazine in unselected IBS would be fruitful.
2. If there is a subgroup that benefit, it is likely to be those with PI-IBS and a trial of such carefully selected patients would be worthwhile, particularly those with more severe diarrhoea.
3. Future work on the role of mast cells needs to better characterise the patients since the majority of unselected IBS do not have elevated mast cell numbers. It may be that as others have reported it is the number of activated mast cells that are important^33^ and better markers of activation would be useful rather than the current gold standard of electron microscopy, which is expensive, time consuming and prone to sampling error.
4. Finally, the release of mediators from biopsies does not link well to symptoms or mast cell numbers. The dominant factor for release is likely to be crushing and tissue injury by the biopsy process that is not well standardised and may overwhelm other factors that would be of more interest. We need a better way of assessing in vivo activity of the mucosal cells.

## Conclusions

This randomised placebo-controlled trial in 115 unselected patients with IBS-D showed that mesalazine 4 g/day was no better than placebo in relieving the symptoms of abdominal pain or disturbed bowel habit. However, contrary to the previous small study (n=10), mesalazine did not reduce mast cell percentage area stained. A small subgroup with PI-IBS appeared to benefit, but this requires a larger adequately powered study to confirm this finding.

Further phenotyping of the heterogeneous group of patients with IBS and diarrhoea is needed to allow better evaluation of new treatments

## Supplementary Material

Web supplement
