# Supplementary material for: A mechanistic multicentre, parallel group, randomised placebo-controlled trial of mesalazine for the treatment of IBS with diarrhoea (IBS-D)
Source: Gut. 2015 Mar 12;65(1):91–9. doi: 10.1136/gutjnl-2015-309122 (PMC4717357; doi:10.1136/gutjnl-2015-309122)
Supplement: Web supplement [file gutjnl-2015-309122-s1.pdf]

Supplementary:

Methodology for immunohistochemical staining of cells for mast cell tryptase, CD3, CD68 and 5-HT (Supplementary table 1).

Biopsies were formalin fixed and embedded in wax prior to standard sectioning for staining.

Supplementary table 1: Summary of antibodies used in immunohistochemistry

**Antibody**	**Supplier (order code)**	**Dilution**	**Pretreatment**
Mast cell tryptase	Dako (M7052)	1/500	• Protease 1 for 4 min<br>• Primary antibody for 32 min<br>• Roche Ultraview detection kit plus Amplification
CD3	Leica (NCL-L-CD3-565)	1/50	• SCC1 (EDTA based buffer) for 64 min<br>• Primary antibody for 32 min<br>• Ultraview detection kit plus Amplification
CD68	Dako (M0814)	1/2000	• SCC1 for 64 min<br>• Primary antibody for 32 min<br>• Ultraview detection
5HT	Dako (M0758)	1/400	• Protease1 for 4 min<br>• Primary antibody for 32 min<br>• Ultraview detection

The slides prepared were scanned into the computer using the nanozoomer and was magnified x40 for ease of portability. Cell counting was performed by a single person (LTX; fellow from the FRAME lab, University of Nottingham) who was blinded to the study. Detection of each stained cell type was checked for reproducibility (>95%) before cell counting began. At least 5-10 areas around lamina propria were drawn and CD68 cells were counted giving an average cell number per mm^2. CD3, which is a marker of lymphocytes, was assessed by counting the number of stained cells at the superficial epithelium per area drawn (mm^2) and an average of obtained. The 5-HT cells were counted at the deep lamina propria and an average of number of cells/mm^2 were obtained. Mast cell tryptase expression was detected in the lamina propria using automatic software (i-Tem by Olympus) as some mast cells may be in a de-granulated state, thus making cell counting difficult. Results were presented as the percentage area stained for mast cell tryptase (%).

Mast cell proteases (tryptase, chymase and CPA3) were measured by sandwich ELISA assays developed by the Immunopharmacology Group, the University of Southampton, as described previously [38-40]. Briefly, antibodies specific for tryptase (EAR), chymase (CC2) and CPA3 (CA2) were coated on 96-well ELISA plates (COSTAR) for 16 h at +4°C. Plates were washed three times and blocked with 2% BSA for one hour at room temperature, and samples or protein standards of tryptase, chymase or carboxypeptidase A3 were added for 90 min. After a further washing stage detecting antibodies specific for tryptase (AA5), chymase (CC5) or carboxypeptidase A3 (CA5) were added, the plates again washed, avidin-horseradish peroxidase added and cleavage of TMB substrate measured colorimetrically at 450 nm. Prior to performing assays, the ELISA was validated for use with cell supernatants, by measuring recovery of each of the proteases spiked into samples prior. Histamine was measured using a commercially available enzyme immunoassay kit (Neogen, Lexington, KY, USA). All assays were performed blind.

Results:

**Supplementary table 2: Baseline characteristics of completers* and non completers**			
**Median (interquartile range)**	**Completers* (n=115)**	**Non-completers (n=21)**	**P value**
Age:	44 (31-54)	47 (42-61)	0.08
Gender (women), N (%)	70 (61%)	12 (57%)	0.81
Anxiety score, mean (SD)	8.7 (4.4)	8.7 (4.5)	0.96
Depression score	4 (2-7)	5 (3-9)	0.27
Patient Health Questionnaire 15	12 (10-15)	13 (8-15)	0.72
Abdominal pain severity (0-10)	4.3 (2.5-5.3)	2.9 (0.9-4.9)	0.10
Urgency (0-10), mean (SD)	5.2 (2.0)	5.1 (1.8)	0.82
Bloating (0-10)	4.4 (2.0-6.1)	2.2 (0.8-6.2)	0.16
Average daily stool frequency	3.4 (2.4-4.6)	2.8(2.2-4.6)	0.37
Average daily stool consistency, mean (SD)	5.4 (0.7)	5.4 (0.8)	0.88
* Completers are defined as those who completed the study and had primary outcome data available at the end of the study.			

Supplementary table 3: Correlation between mast cell percentage area stained with clinical symptoms and biopsy supernatants (N=44)

Symptoms / supernatant values	Spearman, r	P value
Average abdominal pain severity (0-10)	-0.02	0.88
Average bloating score (0-10)	-0.18	0.22
Average urgency score (0-10)	0.67x10^{-}3	0.99
Average stool frequency	0.05	0.74
Average stool consistency	0.15	0.30
Tryptase supernatant (ng/ml)	0.15	0.36
Chymase supernatant (ng/ml)	0.0035	0.98
Carboxypeptidase A3 supernatant (ng/ml)	0.10	0.54
Histamine supernatant (ng/ml)	-0.10	0.55

Stool calprotectin

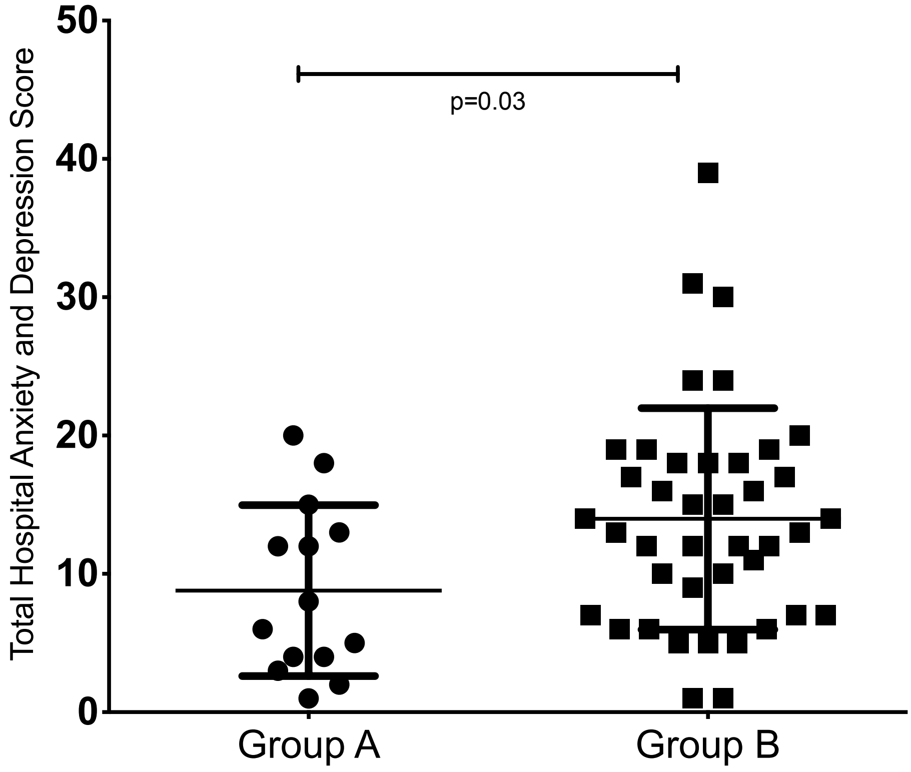

Supplementary figure 1: Baseline total HAD scores showing a significantly lower score in Group A (calprotectin >100ug/g) compared to Group B (calprotectin ≤100ug/g)

PI-IBS

In the post hoc analysis, the subgroup of PI-IBS patients taking Mesalazine had symptom improvement in urgency score and stool consistency (Supplementary figures 2a and 2b).

Supplementary Figure 2a

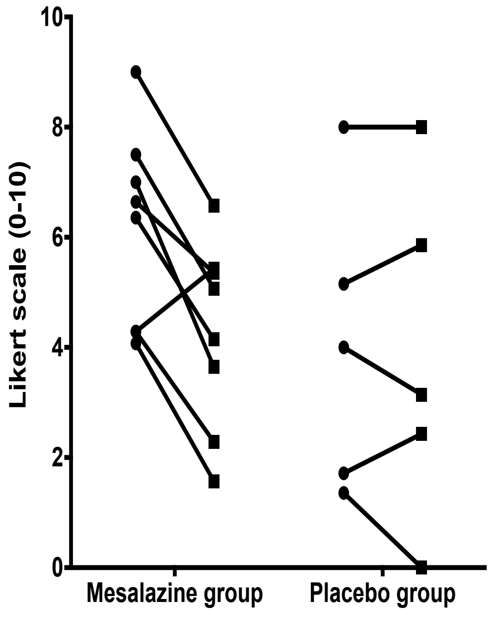

Supplementary Figure 2b

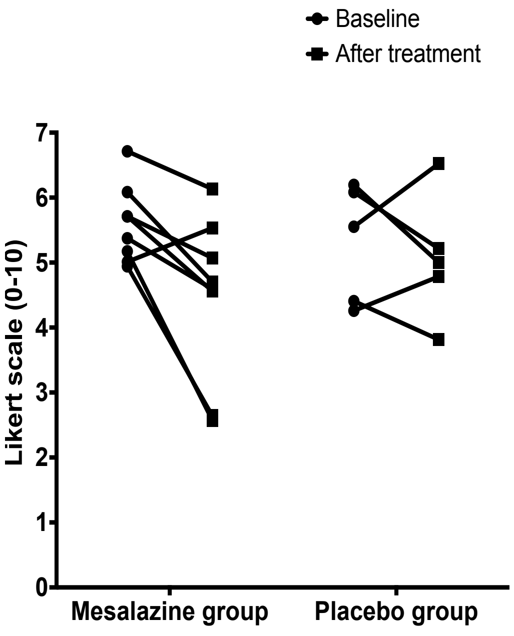

Supplementary figure 2: Urgency score improved following treatment of Mesalazine in PI-IBS patients (Supplementary Figure 2a) and improvement in average stool consistency following treatment of Mesalazine in PI-IBS patients (Supplementary Figure 2b).

**Supplementary table 4: Baseline characteristics for patients who fulfilled the criteria for PI-IBS**		
**PI_IBS (n=13)**<br>**Median (IQR)**	**Mesalazine (n=8)**	**Placebo (n=5)**
Gender, female (%)	4 (50%)	4 (80%)
Age	40 (28-51)	56 (34-63)
Average abdominal pain severity (0-10)	5.4 (4.5-6.1)	2.5 (1.4-5.1)
Average urgency score (0-10)	6.5 (4.3-7.4)	4.0 (1.5-6.6)
Average bloating score (0-10)	3.9 (2.1-6.3)	4.6 (1.6-8.1)
Average daily stool frequency	4.3 (2.7-6.1)	2.3 (2.0-6.7)
Average daily stool consistency	5.5 (5.0-6.0)	5.6 (4.3-6.1)
Total Hospital Anxiety and Depression score	16 (10-28)	10 (5-19)

Compliance

**Supplementary table 5: Compliance with medication during the whole 12 weeks of study**		
	**Mesalazine (n=57)**	**Placebo (n=59)**
**Compliance[1]**<br>**Mean [SD]**	71%[19%]	72%[17%]
**Complier[2]**<br>**N (%)**	33(58%)	35(59%)
[1] calculated as 200 minus medication sachets returned at EOT<br>[2] Complier defined as compliance ≥75%		

Adverse events

Supplementary table 6: Adverse events following randomisation		
**Adverse event**	**Mesalazine**	**Placebo**
Exacerbation of IBS (worsening abdominal pain and/or diarrhoea)	2	3
Bloating	0	2
Dizziness	1	0
Chest pain	1	0
Rash	0	1
Discoloured urine	1	0
Pregnant	1	0
Flu-like illness	1	0
Breast cancer	1	0

Stool diary

Supplementary table 7: Summary of number of days with stool diary entered at baseline and 11-12 weeks	
	**Number of days with stool diary recorded:<br>Mean (SD)<br>Median (IQR)**
Baseline	13.9 (0.3)<br>14 (14,14)
11-12 weeks	13.8 (1.2)<br>14 (14,14)
